# Role of Mitochondrial Stress Response in Cancer Progression

**DOI:** 10.3390/cells11050771

**Published:** 2022-02-23

**Authors:** Yu Geon Lee, Do Hong Park, Young Chan Chae

**Affiliations:** 1 Department of Biological Sciences, Ulsan National Institute of Science and Technology (UNIST), Ulsan 44919, Korea; ugun2@unist.ac.kr (Y.G.L.); dhpark@unist.ac.kr (D.H.P.); 2 Korea Food Research Institute, Wanju 55365, Korea

**Keywords:** mitochondrial protein quality control, mitophagy, mitochondrial dynamics, mtDNA, mitochondrial stress response

## Abstract

Mitochondria are subcellular organelles that are a hub for key biological processes, such as bioenergetic, biosynthetic, and signaling functions. Mitochondria are implicated in all oncogenic processes, from malignant transformation to metastasis and resistance to chemotherapeutics. The harsh tumor environment constantly exposes cancer cells to cytotoxic stressors, such as nutrient starvation, low oxygen, and oxidative stress. Excessive or prolonged exposure to these stressors can cause irreversible mitochondrial damage, leading to cell death. To survive hostile microenvironments that perturb mitochondrial function, cancer cells activate a stress response to maintain mitochondrial protein and genome integrity. This adaptive mechanism, which is closely linked to mitochondrial function, enables rapid adjustment and survival in harsh environmental conditions encountered during tumor dissemination, thereby promoting cancer progression. In this review, we describe how the mitochondria stress response contributes to the acquisition of typical malignant traits and highlight the potential of targeting the mitochondrial stress response as an anti-cancer therapeutic strategy.

## 1. Introduction

**Figure 1:**
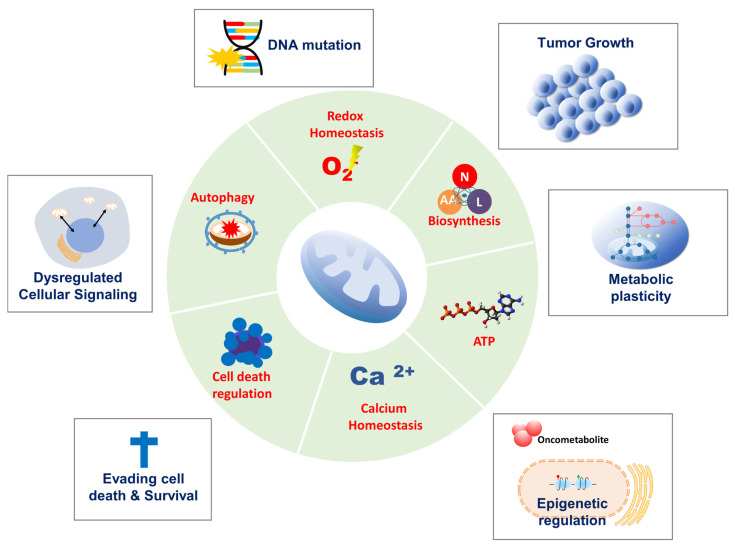
Mitochondria and cancer. Mitochondria play a crucial role in several biological functions, including bioenergetics, biosynthesis, calcium homeostasis, redox homeostasis, autophagy, and cell death regulation. Therefore, mitochondria function is involved in all steps of oncogenesis from tumor initiation to growth, metastasis, and response to treatment.

**Figure 2:**
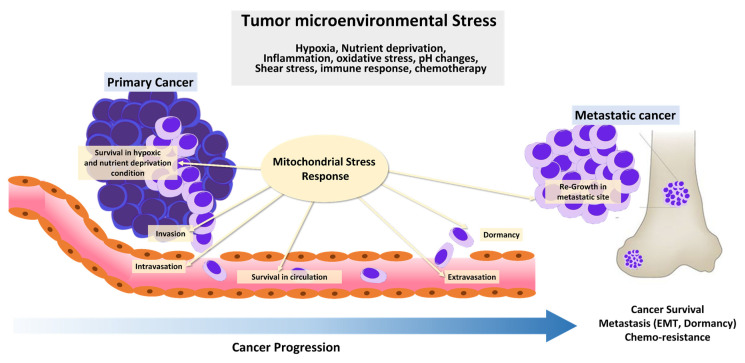
Tumor microenvironmental stress and cancer progression. During cancer progression, cancer cells are constantly exposed to diverse cytotoxic stressors in harsh tumor environments, such as nutrient starvation, hypoxia, oxidative stress, inflammation, oxidative stress, pH changes, shear stress, immune response, and chemotherapy. Stress response activation increases their capacity to survive in environmental conditions encountered during dissemination and confers a survival advantage for tumor growth, metastasis, dormancy, and drug-resistance.

In 1924, Otto Warburg first reported that cancer cells metabolize glucose anaerobically and increase lactate production, even in the presence of oxygen, suggesting that defects in mitochondrial respiration may be the initiating factor in cancer formation [1]. This became known as aerobic glycolysis or the ‘Warburg effect,’ which he interpreted as mitochondrial dysfunction. The Warburg effect provided the rational for the development of a diagnostic tool, ^18^F-2-deoxyglucose accumulation detected by positron emission tomography, which is now used extensively in the clinic for tumor detection and monitoring. Furthermore, the discovery of mutations in mitochondrial enzymes of the tricarboxylic acid (TCA) cycle, such as succinate dehydrogenase (SDH), fumarate hydratase (FH), and isocitrate dehydrogenase 1, and 2 suggests that mitochondrial dysfunction enhances tumor growth or promotes cancer progression. However, the importance of mitochondrial function in cancer has been under-investigated. Recent studies show that cancer cells rely more heavily on mitochondrial functions than previously thought. In contrast to Warburg’s observation that mitochondria are dysfunctional in cancer, mitochondria are metabolically altered to support cancer cell proliferation and tumorigenesis. Mitochondrial function, including oxidative phosphorylation (OXPHOS), is essential for cancer cell viability because the elimination of cancer cell mitochondrial DNA (mtDNA) reduces their growth rate and compromises tumorigenesis. Moreover, most cancer cells exhibit aerobic glycolysis with their mitochondria remaining intact and their respiration rate remaining essentially unchanged from that in normal tissue. Mitochondria not only play a central role in bioenergetics and biosynthesis, but also regulate calcium homeostasis, generation of reactive oxygen species (ROS), production of oncoproteins and oncometabolites, and initiation of programmed cell death (Figure 1). These physiological processes reciprocally affect cancer cell growth by modulating biosynthetic pathways, cell signaling pathways, and transcription factors [1]. Given the importance of mitochondria for vital cellular processes, it is unsurprising that crucial mitochondria functions are implicated in all steps of oncogenesis, from tumor initiation, to growth, metastasis, and response to treatment [2]. To survive hostile tumor microenvironments that perturb mitochondrial functions, cancer cells activate an adaptive mechanism to buffer metabolic and proteotoxic stress. The mitochondrial stress response enables rapid adjustment to the adverse environmental conditions encountered during tumor cell dissemination and confers a survival advantage leading to tumor growth, metastasis, dormancy, and drug resistance [3] (Figure 2). In this review, we provide up-to-date information on mechanisms and functions of the mitochondrial stress response and highlight its therapeutic potential for the suppression of tumor cell growth and survival and prevention of metastasis.

## 2. Mitochondrial Functions in Cancer Progression

### 2.1. Tumor Initiation 

ROS is a common byproduct of OXPHOS that is often elevated due to defective electron transport chain (ETC) activity, which affects redox homeostasis [4]. Mitochondrial ROS production is frequently associated with a shift from cytosolic redox balance to a more oxidized state that, which may contribute to oncogenic transformation, though excessive mitochondrial oxidative stress can trigger cell death in both transformed and non-transformed cells [5,6]. Moreover, excessive ROS can cause DNA mutations, which in turn contributes to genomic instability in various ETC genes [1]. Accumulated mitochondrial oxidative stress can then cause mitochondrial enzyme defects, leading to mitochondrial metabolic reprogramming [7]. Mutations in SDH, FH, and isocitrate dehydrogenases 1 and 2 are frequently observed in a variety of human tumors [8]. These enzymes share metabolic proximity in the TCA cycle. Either gain- or loss-of-function mutations in these key TCA cycle enzymes results in overproduction of the oncometabolites D-2-hydroxyglutarate (D-2HG), L-2-hydroxyglutarate, succinate, and fumarate [9], which alters signal transduction and regulation of gene expression and, thus, promotes malignant transformation and cancer initiation. For example, increased succinate and fumarate stabilize hypoxia-inducing factor (HIF) 1, which induces energy metabolism remodeling under low oxygen conditions and promotes cancer development [10]. In addition, D-2HG, succinate, and fumarate regulate the cancer epigenome. Accumulation of oncometabolites inhibits histone demethylation by competitively inhibiting the catalytic reactions of Jumonji C domain–containing histone lysine demethylases. Moreover, oncometabolites suppress α-KG-dependent nucleotide demethylases, including ten-eleven translocation methylcytosine dioxygenase (TET). Decreased TET enzymatic activity is associated with low 5-hydroxymethylcytosine levels and overall DNA hypermethylation, resulting in expression of a potentially oncogenic transcriptional program [11]. This implies that mitochondrial metabolic alterations, including increased ROS and oncometabolite production, can contribute to malignant transformation. 

### 2.2. Tumor Growth

Cancer mitochondria exhibit enhanced enzymatic activity that stimulates amino acid and fatty acid synthesis [12,13,14,15,16,17,18], which is critical as proteins and lipids are required building blocks that support rapid cell division and membrane integrity [19]. In addition, mitochondria are essential for the synthesis of hormone precursors, such as androgen and estradiol, which are major oncogenic factors in hormone-related malignancies, such as prostate and breast cancer [20,21,22]. Thus, mitochondria dynamically change their function, resulting in highly plastic metabolic rewiring and related genetic alterations during tumor progression. 

Nutrient deprivation and hypoxia during tumor progression are universal phenomena, as most solid tumors outgrow their vascular network and blood supply becomes insufficient [23]. Although cancer cells favor aerobic glycolysis to sustain their proliferative capacity, mitochondria are also essential for replenishing TCA cycle intermediates for the synthesis of nucleotides, amino acids, and lipids under adverse environmental conditions, such as oxygen and glucose limitation [24,25,26]. In this context, mitochondria use metabolites, including lactate, serine, and glycine, as carbon sources if there is insufficient pyruvate from glycolysis [27,28,29]. Mitochondria also utilize fatty acid oxidation as an alternative pathway for energy generation in response to environments unfavorable to glycolysis, such as local acidosis [30]. Under hypoxic conditions, cancer cell mitochondria preferentially activate reductive carboxylation of glutamine metabolism for anaplerotic circuitry of TCA intermediates, which renders cancer cells heavily reliant on the reductive carboxylation of glutamine-derived α-ketoglutarate to generate fatty acids for proliferation [26]. Moreover, reductive glutamine metabolism is required for maintaining reducing equivalent availability to maintain redox balance [31]. Additionally, serine catabolism via serine hydroxymethyltransferase 2 provides reducing equivalents to maintain NADPH/NADP balance [32], whereas increased serine synthesis by phosphoglycerate dehydrogenase leads to increased α-ketoglutarate levels that support anaplerotic flux for cancer growth [33]. Moreover, different tumor cells secrete lactate, which cancer cells absorb and metabolize to fuel OXPHOS in glucose-depleted conditions [34]. In this step, cytosolic lactate is converted to pyruvate by lactate dehydrogenase, which is then imported into mitochondria by mitochondrial pyruvate carriers (MPC) [35]. Mitochondrial pyruvate is then converted to acetyl-CoA by mitochondrial pyruvate dehydrogenase (PDH) [36]. In normal cells, mitochondrial acetyl-CoA is oxidized and primarily used to produce ATP, whereas cancer cells preferentially use acetyl-CoA for the synthesis of fatty acids, isoprenoids, and cholesterol that is necessary for rapid proliferation [1,37]. Thus, cancer cells upregulate pathways that generate acetyl-CoA for lipid synthesis under metabolic stress conditions [38,39]. In addition, MPC-mediated mitochondrial pyruvate oxidation is upregulated and acts as a molecular switch between OXPHOS and glycolysis, providing a proliferative advantage in various cancers, including breast, colon, liver, and prostate cancer [22,40]. These observations indicate that both mitochondrial bioenergetics and precursor production are essential for cancer proliferation. 

### 2.3. Survival and Metastasis

Cancer cells are constantly exposed to cytotoxic stressors, such as nutrient starvation, low oxygen, and oxidative stress in harsh tumor microenvironments. Excess or prolonged exposure to these stressors can cause irreversible mitochondrial damage, leading to cell death [41,42]. In addition, metastatic cells must develop a mechanism to evade cell death caused by various stressors before and after disseminated cells reach a new environment [43]. Therefore, malignant cells acquire several alterations that increase the mitochondrial threshold for maintaining mitochondria integrity and evading cell death.

Hypoxia is a key feature of the tumor microenvironment that affects cell survival and metastasis and has crucial implications for cell signaling pathways [44]. Aberrant cell signaling in cancer allows malignant cells to adapt to hypoxic environments, and their ability to sense and adapt to fluctuations in cellular oxygen levels is highly dependent on HIFs [45]. When oxygen is limited, cancer cells activate HIF-1, which induces multiple oncogenic signaling pathways required for cancer cell survival and tumor progression. Thus, elevated HIF-1 levels are highly correlated with cancer proliferation, angiogenesis, migration and invasion, poor patient prognosis, and therapeutic resistance [46,47,48,49]. Because hypoxic conditions cause mitochondrial respiratory chain complex dysfunction, which leads to excessive ROS production and cell death, cancer cells activate HIF-1 to generate ATP through glycolysis, producing less oxidative stress [50]. Subsequently, HIF-1 increases glucose import and glycolytic rate in cancer cells by activating expression of glucose transporters, hexokinase 2, and pyruvate dehydrogenase kinase 1 [51,52,53,54]. Moreover, HIF-1 reduces mitochondrial activity by decelerating electron transfer from respiratory chain complex 1 and 4 to prevent oxidative stress [55,56]. HIF-1 activation can suppress mitochondria respiration by inhibiting pyruvate dehydrogenase, an enzyme that converts pyruvate to acetyl-CoA [57]. Mutations in TCA cycle genes, such as FH and SDH, results in accumulation of TCA cycle intermediates, including fumarate and succinate, which increases HIF-1 transcription. Moreover, accumulation of fumarate and succinate can inhibit the activity of prolyl hydroxylase, an enzyme that disrupts HIF-1 activity [55,58]. In summary, HIF-1 activation in tumor cells is recognized as a key adaptive mechanism in hypoxic environments, suggesting that environmental stress alters mitochondrial function and thus affects cancer cell metabolism and promote tumor progression.

## 3. Mitochondrial Quality Control in Cancer

**Figure 3:**
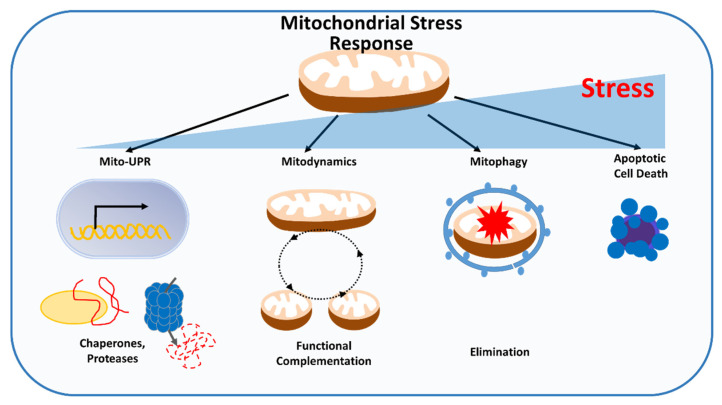
Mitochondrial stress response. Different levels of quality control exist within mitochondria to detect and repair defects that affect mitochondrial performance before reaching the point of inescapable cell death: (Mito-UPR) adjusting protein folding capacity by mitochondrial chaperones and proteases, (Mitodynamics) allowing exchange of material and promoting functional complementation, and (Mitophagy) eliminating damaged mitochondria.

To survive, cancer cells must mitigate the accumulation of mitochondrial damage from environmental stress, which has the potential to perturb mitochondrial and cellular activities. Different levels of quality control mechanisms exist within mitochondria to monitor and repair defects that affect mitochondrial performance before cell death is triggered. The first line of defense occurs on the molecular level and consists of mitochondrial chaperones and proteases that can refold or degrade misfolded or unfolded proteins, alleviating proteotoxic stress. At the organelle level, mitochondrial dynamics allows exchange of material, promote functional complementation and mitophagy, and eliminate damaged mitochondria to ensure mitochondrial quality control. The third level of quality control occurs at the cellular level, whereby extensive mitochondrial damage promotes release of proapoptotic factors, resulting in turnover of the entire cell through apoptosis (Figure 3). 

### 3.1. Mitochondrial Protein Quality Control in Cancer

Mitochondrial stress responses are closely linked to the protein quality control system. Cellular stressors, including oxidative and hypoxic stress, cause protein misfolding and denaturation, which leads to accumulation of protein aggregates in mitochondria. The canonical ubiquitin-proteasome system that is responsible for cytosolic protein homeostasis does not exist in mitochondria. Thus, the mitochondria-specific unfolded protein response (mtUPR) functions to attenuate the accumulation of misfolded proteins in mitochondria. Specialized molecular chaperones and proteases are as a key mtUPR enzymes that clear aberrant proteins. mtUPR increases the proteostatic threshold for adaptation to cytotoxic stressors in cancer cells. 

Accumulating evidence shows that molecular chaperones are overexpressed in tumor mitochondria compared with normal cell mitochondria and may be directly involved in cancer progression. For example, heat shock protein 90 (HSP90) and its homolog TNF receptor-associated protein-1 (TRAP-1) are extensively involved in the mitochondrial chaperone network in tumor cells that controls protein folding quality [59]. The HSP90/TRAP-1 mitochondrial protein folding system is required for tumor survival and to maintain OXPHOS capacity under starvation or hypoxic conditions [60]. In addition, TRAP-1 is expressed more highly in glioma stem cells than in their differentiated counterparts, and TRAP-1 activation promotes cellular metabolism via mitochondrial respiration, which is required for survival under low glucose conditions [61]. Moreover, TRAP-1 induces HIF-1 stabilization and participates in molecular machinery that decreases mitochondrial respiration to confer tumorigenic potential [62]. 

The well-characterized mitoproteases that maintain mitochondrial protein quality control in cancer, lon peptidase 1 (LONP1) and caseinolytic mitochondrial matrix peptidase proteolytic subunit (ClpP), are located in the mitochondrial matrix. Increased LONP1 expression is observed in human colorectal cancer and melanoma and is correlated with poor patient prognosis. LONP1 knockdown causes mitochondrial metabolic dysfunction and reduces tumor proliferation by impairing OXPHOS capacity [63]. Moreover, elevated LONP1 expression is correlated with high glioma tumor grade and poor patient survival, and LONP1 silencing dramatically reduces glioma cell survival under hypoxic conditions [64]. In addition, low oxygen induces LONP1 expression, which maintains oxidative bioenergetics by degrading misfolded ETC subunits, which enhances metastatic competence [65,66]. Comparable to the essential role of LONP1 in maintaining OXPHOS, ClpP deficiency impairs mitochondrial respiration and increases oxidative stress, suppressing cell proliferation and metastatic dissemination [67]. Human acute myeloid leukemia (AML) cell lines lacking ClpP undergo cell death following abnormal protein accumulation and mitochondrial respiration impairment [68]. In addition, ClpP expression is increased in patients with AML, breast, lung, liver, prostate, and thyroid cancer [67,68,69,70,71]. Accordingly, increased ClpP expression is associated with poor outcome and metastasis-free survival in patients with lung, breast, and melanoma [67,70]. We recently that found that LONP1 and ClpP share numerous target substrates that are crucial components of mitochondrial functions, including OXPHOS and amino acid and lipid metabolism, which work cooperatively to maintain protein quality [72]. Indeed, depletion of both genes additively attenuates cancer cell proliferation and mitochondrial bioenergetics, thereby reducing cancer survival during oxidative and metabolic stress [72]. These findings suggest that the capacity of chaperones and proteases to dynamically regulate mitochondrial protein homeostasis provides a high-level quality control system that cancer cells utilize to respond to cytotoxic stress.

### 3.2. Changes in Mitochondrial Dynamics in Cancer

Mitochondria are highly dynamic organelles that have a variety of morphologies, including small spheres, short or long tubules, or interconnected tubules [73]. These morphologies are continuously coordinated by the opposing processes of fusion and fission, which control mitochondria shape, distribution, and size [74,75]. Fusion and fission are highly conserved processes. Fission is orchestrated by mediators dynamin-related protein 1 (DRP1) and mitochondrial fission factor (MFF), whereas fusion is orchestrated by mitofusin (Mfn) 1 and 2 and OPA1 [76]. A critical aspect of mitochondrial dynamics is the selective elimination of mitochondria that are rendered dysfunctional by damaged protein or lipid oxidation and mtDNA mutations, which serves as a quality control mechanism to ensure healthy mitochondrial populations [77,78]. For example, fusion dilutes damaged mitochondrial components, whereas fission segregates depolarized mitochondria, allowing the removal of unhealthy mitochondrial components by mitophagy [77,78]. Mitochondrial dynamics are aberrantly regulated in different types of cancer in response to mitochondrial stress. Consequently, mitochondria use various mechanisms to maintain mitochondrial integrity and support cancer cell survival. 

Fragmented mitochondria with enhanced activation of fission regulators or reduced expression of fusion regulators are frequently observed in various tumor types, including liver, colorectal, brain, lung, and breast cancer [32,79,80,81,82]. Numerous reports demonstrate that oncogenic signaling pathways are required for DRP1-mediated mitochondrial fission. For example, DRP1 is phosphorylated by ERK1/2 on Ser616, which is responsible for enhanced mitochondrial fission and tumor growth in MAPK-transformed tumors, such as melanoma and pancreatic cancer [83,84]. CDK5 also activates DRP1 by phosphorylation, which is correlated with poor outcomes in patients with glioblastoma [83]. PI3K-Akt signaling is closely linked to fragmented mitochondrial networks [85]. In addition, increased DRP1 expression is observed in lymph node metastasis tissue compared with primary tumors [80]. Thus, activation of DRP1-mediated fission is required for tumor migration and metastasis in breast, thyroid, brain, and prostate cancer [80,85,86,87]. 

In hormone-responsive breast and prostate cancer cells, androgens and estradiol influence changes in mitochondrial dynamics [88]. Recently, we investigated the role of androgen-dependent mitochondrial fission on prostate cancer cell survival and found that DRP1 is upregulated by androgen receptor (AR) signaling. Subsequently, DRP1 enhances prostate cancer cell (PCa) proliferation through formation of the voltage-dependent anion channel (VDAC)-mitochondrial pyruvate carrier (MPC) complex to support mitochondrial pyruvate translocation [20]. In addition, DRP1 activation prevents cell death by hypoxic stress, and inhibition of DRP1-mediated mitochondrial fission weakens cell survival under hypoxic and oxidative stress conditions [20]. In another AR-driven PCa model, blockade of mitochondria pyruvate import by MPC inhibition limits metabolic rewiring of the TCA cycle, a hub of bioenergetics and biosynthesis essential for survival [22]. Additionally, the proliferation of androgen-treated PCa is increased by activating autophagy to support adaptation to the tumor environment [89]. Moreover, fragmented mitochondria are frequently found in triple negative breast cancer (TNBC) patient samples and are correlated with poor outcomes [90]. In human breast cancer xenografts, MFN2 downregulation promotes tumor survival and is correlated with an increased risk of cancer-related mortality [91]. DRP1 expression is activated under hypoxic conditions in the MDA-MB-231 TNBC line, but not in the ER-positive MCF7 breast cancer cell line, and decreased mitochondrial fragmentation by DRP1 silencing suppresses TNBC, but not MCF7, cell migration. [92]. Moreover, estradiol-treated MCF7 cells upregulate fusion-related genes, including Mfn 1 and 2, in an ER-dependent manner to increase mitochondrial biosynthesis and cell proliferation [93]. Therefore, oncogenic factors, such as hormones and cell signaling pathways, alter cancer cell mitochondrial dynamics during tumor progression, are crucial for cancer cell survival, and play a context-dependent role in response to stress.

Fission regulation also occurs at the receptor level, involving molecules such as the DRP1 receptor protein MFF. Under energy limited conditions, 5′-AMP-activated protein kinase (AMPK) is activated and can boost mitochondrial bioenergetics [94]. Strikingly, MFF can be phosphorylated by AMPK, after which it enhances mitochondrial fission and increases mitochondrial bioenergetics [95]. We found that MFF is a direct transcriptional target of oncogenic Myc and is overexpressed in primary and metastatic cancer compared with normal tissue, suggesting that increased MFF expression contributes to tumor malignancy [96]. Moreover, MFF complexes with VDAC at the mitochondrial outer membrane (MOM). Disruption of the MFF-VDAC complex by cell-permeable MFF peptidyl mimicry causes acute mitochondrial dysfunction and sudden extensive cell death in various tumor types [97]. MFF is upregulated in metastatic PCa and stem cell enriched tumor spheres compared with primary tumors and normal prostate tissue; moreover, MFF repression limits tumor growth by impairing asymmetric stem cell division with loss of self-renewal [98]. These findings suggest that alterations of mitochondrial dynamics are complex, tightly controlled mechanisms that support cancer progression under stressful conditions. However, more efforts are needed to understand the mechanisms by which tumor cell mitochondrial dynamics respond to environmental stress depending on cancer type or oncogenic factors.

### 3.3. Mitophagy in Cancer

Mitophagy, in which mitochondria are selectively removed by autophagosomes, plays a central role in the elimination of dysfunctional mitochondria and reduction of mitochondrial mass as an adaptive response to environmental stressors such as hypoxia, nutrient deprivation, and DNA damage [99,100]. Thus, when mitophagy machinery is dysregulated, mitochondrial function is impaired and defective mitochondria accumulate, making it difficult for adequate cellular responses to changes in tumor environmental stress [101]. However, excessive mitophagy can cause loss of functional mitochondria resulting in insufficient cellular energy, ultimately leading to cell death [102]. Therefore, mitophagy is tightly regulated and coordinated with other stress response pathways for cell survival in diverse cancers.

Reduced mitochondrial respiration as a result of metabolic stress impairs ATP synthesis efficiency and subsequently activates the AMPK pathway, leading to autophagy initiation [103]. Autophagy initiation is also governed by the unc-51, such as autophagy activating kinase 1 (ULK1, also known as ATG1) complex, which is activated by nutrient-sensing inactivation of mammalian target of rapamycin complex 1 (mTORC1) [104]. mTORC1 is in turn inhibited by AMPK, which also directly catalyzes the activating phosphorylation of ULK1 [104]. Importantly, cells lacking ULK1 cannot be phosphorylated by AMPK and accumulate defective mitochondria under metabolic stress conditions, suggesting that AMPK activates mitophagy in addition to inducing general autophagy through phosphorylation and activation of ULK1 [105]. Thus, AMPK activation can remove damaged mitochondria and inhibit hepatocarcinogenesis in mouse models [105]. Failure to clear damaged mitochondria following mitophagy induces severe oxidative stress-induced cell death by excess lipid peroxidation [106]. In addition, AMPK activation by energy stress induces phosphorylation of MFF, the mitochondrial receptor for DRP1; therefore, mitochondrial fission can contribute to mitophagy induction [95]. Moreover, upstream inhibition of AMPK by liver kinase B1 (LKB1) depletion causes mitochondrial dysfunction, including altered biosynthesis and bioenergetics, and impairs redox balance, thereby promoting tumorigenesis [107]. However, whether the results of deficient AMPK activity are due to changes in mitophagy is not clear. Therefore, further experiments are needed to identify the role of AMPK-driven mitophagy in cancer cell survival. 

PTEN-induced kinase 1 (PINK1)/Parkin-mediated removal of depolarized mitochondria is proposed as a key pathway in mitophagy. In this pathway, PINK1 accumulates on the surface of depolarized and damaged MOM and recruits the E3 ubiquitin protein ligase Parkin to the MOM, where it catalyzes the ubiquitination of mitochondrial proteins, delivering mitochondria to the autophagosome [108,109]. Recent evidence suggests that Parkin improves mitochondrial integrity by increasing oxidative metabolic levels and inhibiting anaerobic glycolysis downstream of p53 tumor suppressor [110]. Parkin can directly bind and ubiquitinate MFN2, promoting melanoma tumor formation and metastasis [111]. PINK1 silencing impairs mitochondrial membrane integrity and causes ROS overproduction, which reduces lung cancer cell migration and invasion capacity [112]. In addition, Parkin-deficient breast cancer cells exhibit reduced proliferation rates and decreased metastatic potential [113]. 

Parkin-independent mechanisms also play a key role in mitophagy. NIX, BNIP3, and FUNDC1 are major receptors for mitophagy on the MOM that recruit autophagosomes to mitochondria under stress conditions, such as hypoxia [114,115]. NIX-mediated mitophagy is highly activated in hypoxic region of glioblastoma, and NIX inhibition impairs mitochondrial ROS clearance and cancer stem cell maintenance, which attenuates cancer cell survival under hypoxic stress [116]. Moreover, loss of NIX delays pancreatic cancer proliferation by impairing mitochondrial redox homeostasis in glycolysis-dependent pancreatic ductal adenocarcinoma [117]. Inhibition of BNIP3-mediated mitophagy markedly reduces adenoid cystic carcinoma cell invasion under low oxygen conditions [118]. Furthermore, hypoxia-induced BNIP3 and NIX co-upregulation promotes mitophagy to support lung cancer cell proliferative capacity following treatment with antitumor drugs, such as cisplatin [119]. Similarly, FUNDC1 is more highly expressed in cancers such as cervical and colorectal cancer than in adjacent normal cells [120,121]. Depletion of FUNDC1 inhibits cell proliferation and increases tumor cell sensitivity to anticancer drugs, including cisplatin [120]. 

**Figure 4:**
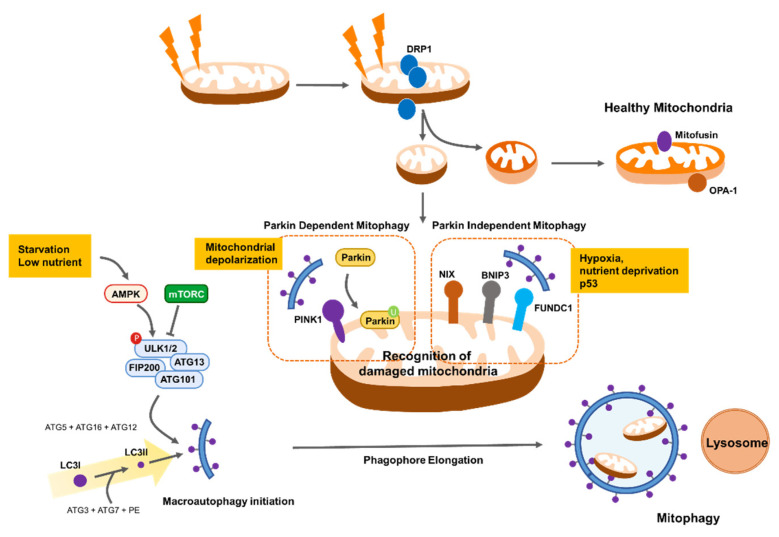
Mitophagy pathway for the survival of cancer cells under diverse stresses. Mitochondrial quality control involves fission and mitophagy to eliminate defective mitochondria. Mitochondrial quality control is a key process for coping with low nutrients and oxygen levels (i.e., hypoxia) in the tumor microenvironment.

Notably, mitophagy is closely involved in mitochondrial fission/fusion because fission enables segregation of depolarized mitochondria from the mitochondrial network and allows their degradation by autophagosomes [78]. Thus, mitochondrial fragmentation may be a prerequisite for mitochondrial degradation by mitophagy. Accordingly, stress-induced mitophagy is frequently accompanied by enhanced mitochondrial fission. Decreased mitochondrial fission, by DRP1 inhibition, suppresses mitophagy, leading to the accumulation of oxidized mitochondrial proteins [78]. In addition, Parkin can increase mitochondrial fission by promoting MFN1 and 2 degradation [122] and prevents mitochondrial fragmentation by ubiquitination of DRP1 [123]. At the MOM, mitophagy receptors, including FUNDC1 and BNIP3, enhance mitochondrial fission in response to metabolic stress [124,125]. Additionally, FUNDC1 directly interacts with DRP1 to coordinate mitochondrial fission and mitophagy and thereby adapt to stressful environments. [125]. Taken together, mitochondrial dynamics and mitophagy are complementary mechanisms that prevent loss of mitochondrial function in response to cytotoxic stress and are key for cancer cell survival (Figure 4). 

### 3.4. Cell Death Regulation

Mitochondria are key regulators of programmed apoptotic and necrotic cell death. Although apoptosis and necrosis proceed through distinct pathways, their molecular mechanisms overlap [126,127]. Thus, they can be activated simultaneously, are reciprocally affected, and can work together in response to severe environmental stress to modulate cell death [127]. Importantly, cancer cells must overcome a requisite mitochondrial threshold to initiate cell death. That is, when stress exceeds the threshold at which mitochondrial membrane integrity can be maintained, the mitochondrial membrane is permeabilized, leading to induction of downstream targets of mitochondrial cell death [128,129,130]. Thus, cancer mitochondria raise the cell death threshold by modulating the stress response pathway and survival-promoting factors, including members of the B-cell lymphoma 2 (BCL-2) family, to protect cancer cells from stress-induced death [131]. Given that the mitochondrial stress response pathway serves as a quality control system by eliminating or restoring unhealthy sectors in the mitochondrial network, it is not surprising that the interplay between stress response pathways and cell death regulators confers a survival advantage for cancer cells.

BCL-2 family proteins are crucial regulators that sense apoptotic stress and ultimately induce mitochondrial outer membrane permeabilization (MOMP), which leads to release of cytochrome c and other apoptotic factors [132]. The BCL2 protein family consists of anti-apoptotic proteins (BCL2, BCL-XL, MCL1) and proapoptotic proteins (BAX, BAK) and their upstream effectors, BH3-only pro-apoptotic proteins (BAD, BIM, PUMA, BID, NOXA) [133]. The balance between anti-apoptotic and pro-apoptotic proteins is determined by a network of physical interactions focused on the BH3 domain [126]. BH3-proteins, including BAD and NOXA, decrease mitophagy by disrupting the anti-apoptotic-BCL-XL complex [134]. Moreover, mitophagy is antagonized by anti-apoptotic BCL-2 proteins (BCL-XL and MCL-1) that bind and prevent translocation of Parkin to depolarized mitochondria, whereas BH3-only proteins enhance Parkin translocation to mitochondria [135]. 

Several molecular chaperones are upregulated by stress-induced mitochondrial damage and mtDNA depletion to prevent cell death. An accumulating body of evidence shows that molecular chaperones, including HSPs, have crucial properties involved in regulation of the apoptosis pathway. For example, HSP60 interacts with pro-apoptotic BAX, and decreased HSP60 expression increases BAX mitochondrial accumulation, leading to cytochrome c-mediated caspase activation [136]. Moreover, overexpression of HSP10 induces accumulation of anti-apoptotic BCL-XL and BCL-2 in doxorubicin-treated cells, due to the reduction of apoptosis-promoting BAX [137]. In addition, stress-induced HSP90 exhibits anti-apoptotic properties by inhibiting the cytochrome c-mediated apoptotic protease cascade [138]. Moreover, HSP90 and HSP60 bind to cyclophilin D, the mPTP component. Disruption of this complex inhibits tumor growth and caspase-dependent apoptosis [139]. HSP60 inhibits the pro-apoptotic function of p53 in cancer cells under apoptotic stimuli by forming a complex with p53 that destabilizes p53 [140]. Thus, increased HSP60 enhances tumor growth and metastatic capacity in various cancer types [141]. HSP27 overexpression also delays caspase activation and decreases sensitivity to etoposide-induced cytotoxicity in human leukemic cells [142]. Increased HSP70 prevents stress-induced cell death by inhibiting mitochondrial BAX translocation and mPTP opening [143]. In addition, TRAP1 expression is decreased by treatment with an apoptosis inducer, whereas TARP1 silencing increases cancer cell sensitivity to oxidative stress [144]. TRAP1 is phosphorylated by PINK1, which delays release of cytochrome c and thereby protects cells from oxidative stress-induced cell death [145]. These findings show that molecular chaperones not only play an important role in protein homeostasis, but also interact with mitochondrial proteins to regulate cell death. 

BCL-2 family proteins move dynamically in the MOM [146]. Under cytotoxic stress, BCL-2 proteins permeabilize the MOM, which is often accompanied by mitochondrial fragmentation [147]. In addition, BAX colocalizes with DRP1 at mitochondria scission sites, and DRP1 inhibition not only decrease mitochondrial fission, but also suppresses caspase-dependent cell death [148]. Indeed, in DRP1-deficient mice, mitochondria are not properly distributed within cells and fail to drive the regulated cell death program during neural tube formation [149,150]. Thus, mitochondrial fission is proposed to be an essential step in the cell death process [73]. However, embryonic fibroblasts lacking DRP1 have similar sensitivity to apoptotic stimuli compared to control fibroblasts [149,150]. Importantly, recent studies show that dysregulated mitochondrial fission activates well-organized stress responses, including autophagy, to protect cancer cells from stress-induced cell death. For example, prostate cancer DU145 and PC3 cells lacking MFF activate the AMPK signaling pathway, leading to autophagy [96]. In addition, DRP1 knockdown induces metabolic stress-induced autophagy in prostate cancer LNCaP cells, and inhibition of autophagy by gene silencing or pharmacological inhibition enhances caspase-dependent cell death by DRP1 depletion [20]. Moreover, cells lacking MFN2 exhibit fragmented mitochondria and do not exhibit cell death induced by apoptotic stimuli, whereas restoration of fused mitochondria by fission inhibition induces necrotic cell death [151]. Additionally, transplantation of healthy mitochondria into TNBC cells downregulates DRP1 and consequently upregulates mitochondrial fusion, resulting in increased oxidative stress, induction of necrosis, and increased susceptibility to chemotherapy [152,153]. These observations are consistent with the fact that fission does not induce cell death per se, implying that a fragmented mitochondrial network increases the mitochondrial threshold for cell death, thereby promoting tumor expansion, metastasis, and drug resistance.

## 4. Mitochondrial DNA Homeostasis in Cancer

Mitochondria contain multiple copies of circular DNA consisting of a total of 16,569 bp. mtDNA encodes 13 ETC subunits, 2 mitochondrial rRNAs, and 22 tRNAs [154]. Although mtDNA only encodes 13 proteins, these proteins are crucial for regulation of mitochondrial functions, including OXPHOS. Removal of mtDNA from a variety of cancer cells confirms the importance of functional mitochondria in cancer cells. Cells depleted of mtDNA (ρ0 cells) via ethidium bromide show delayed tumor initiation and metastasis. Tumorigenic potential can be restored in ρ0 cells by adding healthy mitochondrial fraction, which increases mitochondrial respiratory activity [155,156]. In addition, mtDNA transfer can allow cancer cells to escape from therapy-induced dormancy [157]. Mitochondrial function and mtDNA integrity are closely related. Oxidative damage and mtDNA replication errors are a major cause of mtDNA mutations. Because the mitochondrial stress response involves mitochondrial dynamics and mitophagy, these processes may contribute to the maintenance of mtDNA and functionally active mitochondria. Under stressful conditions, mitochondria can change number and shape by continuous fission and fusion processes in different cell types to maintain their integrity and selectively degrade damaged mtDNA, eliminating accumulated mtDNA mutations [158,159]. Thus, when mitochondria lose their ability to respond to severe stress, the proportion of mutant mtDNA increases and produces bioenergetic defects ranging from mild mitochondrial dysfunction to severe metabolic disorders and cell death [160]. Deficiency of mitochondrial fusion factor leads to mtDNA copy number reduction and accumulation of point mtDNA mutations, causing mitochondrial dysfunction [161]. Moreover, reduced mitophagy can lead to increased accumulation of mutated mtDNA and dysfunctional mitochondria. These findings suggest that sufficient mitochondrial DNA homeostasis plays a crucial role in tumorigenesis and tumor survival during dissemination and metastasis. In addition, major factors involved in mtDNA homeostasis include mtDNA repair and replication, mtDNA copy number alteration, mtDNA mutations, and regulation of transcription and translation of mtDNA. 

### 4.1. Mitochondrial DNA Mutation and Copy Number in Cancer

Recent evidence shows that a variety of cancers involve many germline and somatic mtDNA mutations associated with cancer risk. Patterns of mtDNA alteration and specific major mutations are cancer and tissue type dependent [162,163]. For instance, mtDNA is most abundant in ovarian cancer (median: 644 copies per cell) and least abundant in myeloid cancer (median: 90 copies per cell). mtDNA copy number analysis comparing tumor and adjacent matched normal tissue shows that mtDNA levels are decreased in tumors relative to matched normal tissue in seven of 15 tumor types, including bladder, breast, and kidney cancer, and are only increased in lung adenocarcinoma [164]. mtDNA copy number is generally decreased in tumor tissue compared with normal tissue, indicating that maintenance of sufficient mtDNA copy number for minimal cell respiration is important for tumorigenesis and metastasis. For example, ρ0 cells exhibit in vivo tumor growth and tumorigenesis step (e.g., primary, circulation, and metastasis) delays of more than 3 weeks. Furthermore, ρ0 cells gain mitochondrial genome from the host depending on the tumorigenesis step. This pattern is correlated with restored mitochondrial respiratory function and shRNA knockdown the mitochondrial complex subunits (NDUFV1, SDHC) similarly decreases tumorigenic ability [165]. In addition, transfer of host-derived mtDNA via extracellular vesicles to hormone therapy-resistant breast cancer helps restore OXPHOS and facilitates dormancy escape of therapy-induced breast cancer stem-like cells [157]. Furthermore, a meta-analysis reports positive correlations between mtDNA content and cancer risk in lymphoma, breast cancer, and colorectal cancer patients [166]. However, some studies report a correlation between decreased mtDNA copy number and elevated cancer cell metastasis and stemness; therefore, more studies on the role of mtDNA copy number alterations for each tumor type are needed [167]. Similar to mtDNA copy number alterations, many specific mutations are found in mtDNA, and the role of mtDNA mutations in tumor malignancies is being actively studied [168]. Each cancer type has a different mutated region in which varying proportions of specific mtDNA mutations are found. Notably, mtDNA mutations in most cancers are clearly heteroplasmic. However, most mutations are single-nucleotide variants (SNVs), which are often not as deleterious as insertions or deletions [162]. In a report detailing mtDNA mutations in breast cancer, somatic mutations were found in 74% of patients, of which most (81.5%) were D-loop mutations occurring at a control region of mtDNA. The remaining mutations (18.5%) were detected in 16S rRNA, ND2, and ATPase 6 genes [169]. In another study, 96.6% of mtDNA variants in breast tumors were SNVs, whereas 3.4% were small deletions and small insertions.

These variants are distributed in both non-coding regions (38.2%) and coding regions (61.8%). Of the variants in the coding regions, 33.3% are synonymous mutations and 66.7% are non-synonymous mutations [170]. mtDNA mutations are not randomly occurring. In cancer cells, mtDNA mutations appear to be regulated by mtDNA repair of severely damaging mutations, except for oncogenic mutations, and these functional mtDNA mutations can adapt tumor energy metabolism to oncogenic conditions [171]. A nearly homoplasmic truncating mutation is observed in kidney cancer mtDNA, suggesting that mitochondrial dysfunction is a fundamental step in kidney tumorigenesis [162]. Moreover, multiple large-scale deletions, including common 4977-bp mtDNA deletions, are highly accumulated in aging tissues. However, the number of mtDNA deletions in most tumors is dramatically less than that in adjacent normal tissue in the same patients [172]. The low frequency of large-scale deletion of tumor mtDNA may be because these mutations cause mitochondrial dysfunction and sensitize tumors to apoptosis. Therefore, cancer cells with many large-scale mtDNA mutations may be removed during cancer initiation and early tumor progression [173]. Due to the heterogeneous and tissue-specific nature of mtDNA, such as mtDNA copy number alternations, further research is needed to determine a causative, cancer-driving role of mtDNA mutations [174].

### 4.2. Mitochondrial DNA Transcription and Translation

In addition to mtDNA copy number alterations, mitochondrial gene regulation is necessary for controlling mitochondrial energy metabolism, which enables cells to adapt to harsh environmental changes [175]. The mitochondrial genome is highly condensed and undergoes multiple processing steps, including chemical modification, after RNA transcription in the light and heavy strands before it is finally assembled into mitoribosomes to translate mitochondrial RNA to proteins [176]. The major machinery of mtDNA transcription includes mitochondrial transcription factor A (TFAM), mitochondrial RNA polymerase (POLRMT), and mitochondrial transcription factors B1 and B2 [177]. As it is widely reported that mitochondrial function is essential in cancer [1], the role of mitochondrial genome transcription in cancer progression is being actively studied. For example, downregulation of TFAM in lung cancer cells leads to G1 arrest and substantial cell growth inhibition through ROS-induced JNK/38 MAPK signaling. In addition, TFAM inhibition increases apoptosis and sensitivity to cisplatin, suggesting that mtDNA transcription could be an important target for non-small cell lung cancer treatment [178]. Screening has identified inhibitors that specifically target human mitochondrial RNA polymerase (POLRMT), which is essential for OXPHOS system biogenesis. For instance, IMT1 inhibits mtDNA expression and OXPHOS in a dose-dependent manner in normal tissue and tumor xenografts. Whereas cancer cell proliferation is strongly inhibited in tumor xenografts, no toxicity is induced in normal tissue [179]. The antitumor effects of inhibitors of mitochondrial transcription are circumvented by increasing mtDNA gene expression or creating more cellular metabolites in the tumor both in vitro and in vivo. This resistance is impaired by inhibiting mitochondrial transcription factor A downregulation and mitochondrial translation [180]. 

These findings suggest that mitochondrial transcription and translation are essential for energy metabolism and tumor progression. However, the importance of mitochondrial transcription in cancer does not mean that mitochondrial RNA levels are higher in tumors than in normal tissue. Mitochondrial copy number is reduced in most solid tumor types [164], and orthogonal RNA sequencing shows that mitochondrial RNA levels are lower in most cancer types including breast, esophageal, head and neck, kidney clear cell, and type of liver cancer compared with normal tissue. Even lung adenocarcinoma, which has an increased mtDNA copy number compared with normal tissue, exhibits lower expression of six out of 13 mitochondrial RNAs. Increased mtDNA copy number in cancer may compensate for low mitochondrial RNA transcript levels [181]. 

Just like mitochondrial RNA transcription, abnormal translation of mitochondrial encoded proteins, which are part of the OXPHOS system, are strongly associated with cancer progression. Mitochondrial translation consists of initiation, elongation, termination, and ribosome recycling stages, during which dysfunction of protein translation factors and translation activators due to mutation or deletion cause various mitochondrial diseases, including cancer [182]. Targeting mitochondrial DNA polymerase or mitochondrial ribosomes is an off-target effect of antibiotics, such as tigecycline, because mitochondria originated from an endosymbiotic bacterium [183]. Tigecycline is an effective therapeutic for leukemic cells, as it selectively kills leukemia stem and progenitor cells. The importance of mitochondrial translation in cancer was confirmed by demonstrating antileukemia activity following genetic inhibition of EF-Tu, a mitochondrial translation factor [184]. Furthermore, knockout of mitochondrial elongation factor 4 (mtEF4), which is responsible for the quality control of respiratory chain biogenesis, causes respiratory chain complex defects and cancer cell apoptosis. mtEF4 overexpressing tumors exhibit enhanced cancer progression through lowered cellular redox [185]. Although mitochondrial RNA expression is reduced in most tumors, inhibition of mitochondrial transcription and translation may severely affect cancer progression and metabolism. These findings suggest that these vulnerabilities can be exploited to develop cancer treatment strategies for certain tumor types.

## 5. Conclusions

It is now recognized that functional mitochondria are crucial for all aspects of cancer. In particular, mitochondrial stress response upregulation to maintain mitochondrial homeostasis supports the metabolic needs of cancer cells and provides a survival advantage required for cancer progression. Therefore, there is great promise in further understanding the molecular mechanisms of mitochondria homeostasis as targeting the mitochondrial stress response, including protein quality control, mitochondria dynamics, mtDNA maintenance, transcription, and translation, and may be a clinically relevant anticancer strategy.
